# Ahnak functions as a tumor suppressor via modulation of TGFβ/Smad signaling pathway

**DOI:** 10.1038/onc.2014.69

**Published:** 2014-03-24

**Authors:** I H Lee, M Sohn, H J Lim, S Yoon, H Oh, S Shin, J H Shin, S-H Oh, J Kim, D K Lee, D Y Noh, D S Bae, J K Seong, Y S Bae

**Affiliations:** 1 Department of Life Sciences and GT5 program, Ewha Womans University, Seoul, Korea; 2 Laboratory of Developmental Biology and Genomics, College of Veterinary Medicine, Seoul National University, Seoul, Korea; 3 College of Pharmacy, Gachon University, Incheon, Korea; 4 Department of Surgery, School of Medicine, Seoul National University, Seoul, Korea; 5 Department of Obstetrics and Gynecology, Samsung Hospital, SungKyunKwan University, Seoul, Korea

## Abstract

We provide detailed mechanisms of Ahnak-mediated potentiation of transforming growth factor β (TGFβ) signaling, which leads to a negative regulation of cell growth. We show that Smad3 interacts with Ahnak through MH2 domain and that Ahnak stimulates Smad3 localization into nucleus leading to potentiating TGFβ-induced transcriptional activity of R-Smad. Moreover, overexpression of Ahnak resulted in growth retardation and cell cycle arrest through downregulation of c-Myc and cyclin D1/D2. We describe results from analyses of Ahnak^−/−^ mouse model expressing middle T antigen in a mammary gland-specific manner (MMTV^Tg/+^Ahnak^−/−^), which showed significantly progressed hyperplasia of mammary glands compared with MMTV^Tg/+^Ahnak^+/+^. Finally, we screened multiple human breast cancer tissues and showed that the expression of Ahnak in cancer tissues is lower than that in control tissues by 50%. Taken together, these data indicate that Ahnak mediates a negative regulation of cell growth and acts as novel tumor suppressor through potentiation of TGFβ signaling.

## Introduction

Ahnak is a protein originally identified as a nuclear phosphoprotein in human neuroblastomas and skin epithelial cells.^1, 2, 3^ A protein of exceptionally large size, Ahnak can be divided into three structurally distinct regions: the amino-terminal 500 amino acids, a large central region of about 4388 amino acids composed of 36 repeat units and the carboxyl-terminal 1003 amino acids. Ahnak may have a role in cardiac calcium signaling by modulating the L-type channel in response to β-adrenergic stimulation in cadiomyocytes.^4,5^ Ahnak protein also interacts with S100B, a calcium-binding protein.^6^ Importantly, it has been reported that Ahnak protein binds and activates phospholipase C-γ1 in the presence of arachidonic acid.^7, 8, 9^ Moreover, Ahnak apparently interacts with protein kinase C (PKC) resulting in regulation of smooth muscle cell migration.^10^ Thus, Ahnak appears to function as a molecular linker for calcium homeostasis in response to agonists.

Transforming growth factor β (TGFβ) is a group of multifunctional cytokines that affect cell growth, cell death, differentiation, apoptosis and tumorigenesis.^11, 12, 13^ TGFβ transduces signals via heteromeric complex of type II and type I serine/threonine kinase receptors. TGFβ type II receptor phosphorylates serine and threonine residues in TGFβ type I receptor (TGFβRI), which results in activation of the type I receptor. Activated type I receptor transduces signals to the cytoplasm through phosphorylation of receptor-regulated Smads (R-Smads). Phosphorylated R-Smads bind to Smad4, a common partner Smad (co-Smad), and translocate into the nucleus, and this complex functions in transcriptional regulation. Many studies have been focused on understanding how TGFβ signals modulate cell cycle.^12,13^ An important event in the TGFβ signaling is the inhibition of c-Myc expression. TGFβ inhibits c-Myc and cyclin D protein expression leading to inhibition of cyclin-dependent kinase (CDK) activities that drive the progression through G1 phase of the cell cycle. In epithelial cells, TGFβ rapidly elevates the expression of CDK4/6 inhibitor p15^Ink4B^. Binding of CDK4 with p15^Ink4B^ in turn inhibits the kinase resulting in induction of cell cycle arrest.

Here we show that Ahnak functions as an important mediator of TGFβ signaling that leads to cell cycle arrest. Detailed mechanistic studies show that TGFβ-induced nuclear translocation of Ahnak leads to potentiation of R-Smad function and thereby downregulation of c-Myc and cyclin D1/D2 as well as inhibition of cell growth. We also provide evidence supporting the novel role of Ahnak using a transgenic mouse model and human cancer samples.

## Results

### Smad proteins that are identified as a binding partner of Ahnak

**Figure 1:**
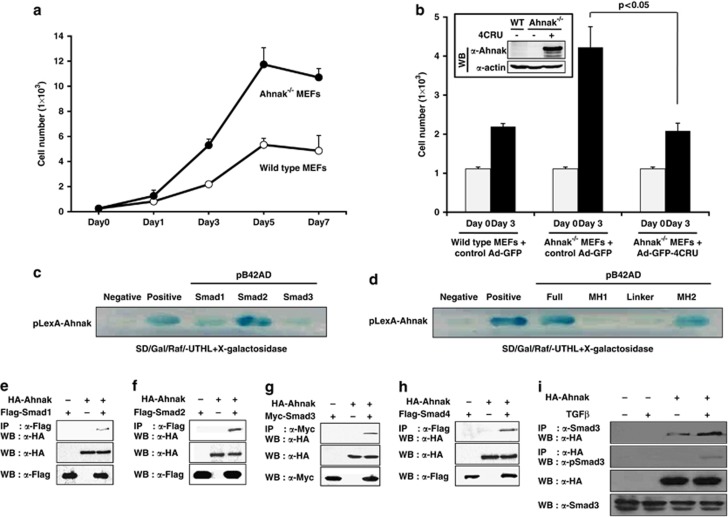
Interaction of Ahnak with R-Smad. (**a**) Growth rate of MEFs from wild-type and Ahnak^−/−^ mice. Both cells (2.5 × 10^3^) were cultured in 96-well plates for the indicated number of days. The cells were harvested and counted. The data are means±s.e.m. of three independent experiments. (**b**) Add-back expression of adenovirus containing GFP-conjugated 4CRU of Ahnak. Wild-type (WT) and Ahnak^−/−^ MEF cells with or without adenovirus containing GFP-conjugated 4CRU (3 × 10^3^) were cultured in 96-well plates for the indicated number of days. The cells were harvested and counted. The data are means±s.e.m. of three independent experiments (*P*<0.05). Infection of adenovirus containing GFP-4CRU of Ahnak was confirmed by immunoblot analysis (inset). (**c**) Yeast cells (EGY48/p80-lacZ) were transformed with one of following plasmids: (1) pLexA-CRU (amino-acid residues 4105–4633) of Ahnak/pB42AD (negative control), (2) pLexA-53/pB42AD-T (positive control), (3) pLexA-Smad1, pLexA-Smad2 or pLexA-Smad3. The transformants were streaked on SD/Gal/Raf/Ura^−^Trp^−^His^−^Leu^−^(UTHL) plate to test Leu2 reporter gene expression. (**d**) Yeast cells (EGY48/p80-lacZ) were transformed with one of the following plasmids: negative and positive control are same as **c**. pLexA-full length (amino-acid residues 2–468), MH1 domain (amino-acid residues 2–183), linker region (amino-acid residues 180–273) and MH2 domain (amino-acid residues 269–468) of Smad2. The transformants were streaked on SD/Gal/Raf/Ura^−^Trp^−^His^−^Leu^−^(UTHL) plate to test Leu2 reporter gene expression. (**e**-**h**) COS7 cells were transfected with Hemaagglutinin (HA)-tagged 4CRU of Ahnak and Flag-tagged Smad1, Flag-tagged Smad2, Myc-tagged Smad3 or Flag-tagged Smad4. Cell lysates were immunoprecipitated (IP) with antibodies to Flag or Myc and immunoblotted with antibody to HA for detecting HA-4CRU of Ahnak-bound Smads. (**i**) NIH3T3 cells were transfected with pcDNA3-HA or pcDNA3-HA-4CRU of Ahnak and treated with 10 ng/ml of TGFβ. The cell lysates were subjected to IP with anti-Smad3 antibody and analyzed by immunoblotting with the indicated antibodies. WB, western blot.

Ahnak-deficient (Ahnak^−/−^) mice showed a stunted growth and reduced adipose tissue, suggesting a complex physiological effect of the loss of Ahnak expression (Supplementary Figure 1 available online). Interestingly, the proliferation rate of Ahnak^−/−^ mouse embryonic fibroblast (MEF) cells was higher than that of wild type (Figure 1a). These phenotypes indicate a complex effect of Ahnak *in vivo*. Several studies have reported that the central repeated units (CRUs) of Ahnak protein serves as scaffolding motif for cell signaling.^8, 9, 10^ We introduced a construct expressing four CRUs (4CRU, amino-acid residues 4105–4633) into MEF cells from wild-type and Ahnak^−/−^ mice and analyzed cell proliferation rate. Ahnak^−/−^ MEFs showed an increased growth rate, whereas add-back expression of 4CRU of Ahnak resulted in a significant decrease to the level of wild-type MEFs (Figure 1b). The result suggested that CRU of Ahnak has a critical role in cell growth. To identify the function of Ahnak proteins in cell growth, we first sought to isolate proteins interacting with Ahnak using the yeast two-hybrid assay. Here, we describe results obtained from the screen using one CRU of Ahnak (amino-acid residues 3988–4129) as the bait. HeLa complementary DNA (cDNA) library (approximately 2 × 10^6^ independent clones) was screened, and about 500 clones were obtained. These clones were grown on selective media and on the X-gal-containing culture plates to assay for β-galactosidase activity. From randomly selected 100 positive clones, 21 genes were isolated (Supplementary Table S1). Notable among them was Smad1, and additional yeast two-hybrid assays showed that Ahnak interacts with all three R-Smads (Smad1, 2 and 3; Figure 1c). To determine which region of R-Smad interacts with 4CRU of Ahnak, we established pB42AD-full length, -MH1 domain, -linker region and -MH2 domain of Smad2 and tested the interaction using yeast two-hybrid assay. As shown in Figure 1d, 4CRU of Ahnak interacts with MH2 domain, but not with MH1 or linker region suggesting that Ahnak binds to Smad isoforms through the MH2 domain, highly conserved among R-Smads and co-Smad.^12,13^ We next investigated whether Smad proteins interact with 4CRU of Ahnak in cell. COS7 cells were transfected with pcDNA3-HA-4CRU along with pCS4-3Flag-Smad1, pCS4-3Flag-Smad2, pCS-Myc-Smad3 or pCS2-Flag-Smad4. The cell lysates were subjected to co-immunoprecipitation with antibody to Flag or Myc and the immune complexes were analyzed with antibody to HA. All tested R-Smad proteins interacted with 4CRU of Ahnak (Figures 1e–h). These results show that Ahnak interacts with Smad proteins and likely acts as a molecular linker in complexes between R-Smads. To investigate whether the 4CRU of Ahnak interacts with endogenous Smad3, we performed co-immunoprecipitation in NIH3T3 cells transiently expressing pcDNA3-HA, or pcDNA3-HA-4CRU of Ahnak. The result indicated that 4CRU of Ahnak interacted with endogenous Smad3 and the interaction was increased by stimulation of TGFβ (Figure 1i).

### Function of Ahnak in the activation of Smad-binding element (SBE) in response to TGFβ

**Figure 2:**
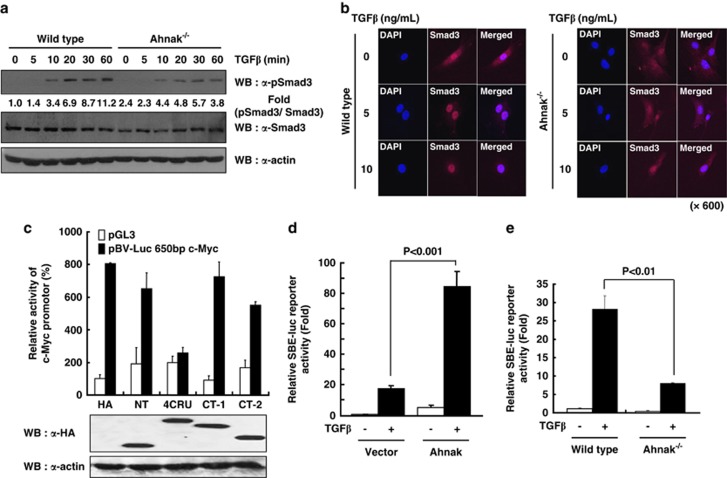
Ahnak potentiates the activation of Smad3. (**a**) MEF cells from wild-type or Ahnak^−/−^ mice were stimulated with TGFβ (10 ng/ml) for the indicated time. Cell lysates were then subjected to western blot (WB) analysis with antibodies to phospho-Smad3 (pSmad3), Smad3 and actin. (**b**) MEF cells from wild-type and Ahnak^−/−^ mice were stimulated with 10 ng/ml TGFβ for 1 h and then cells were fixed and stained with antibody to Smad3. Tetramethylrhodamine isothiocyanate (TRITC)-conjugated goat anti-mouse IgG was used as the secondary antibody for staining. 4'-6-Diamidino-2-phenylindole (DAPI) indicates nuclear staining. (**c**) Effect of various domains of Ahnak on c-Myc expression. NIH3T3 cells were co-transfected with reporter construct containing c-Myc promoter region (pBV-Luc 650bp c-Myc; -1073 to -423) and various domains of Ahnak (NH2-terminal, 4CRU, COOH-terminal 1 and COOH-terminal 2) or control vector. Luciferase activity assay was described in Materials and methods. The cell lysates were subjected to immunoblot analysis with antibodies against HA and re-probed with antibody to actin (lower panel). (**d** and **e**) NIH3T3 cells (**d**) and MEF cells from wild-type and Ahnak^−/−^ mice (**e**) were transfected with pSBE-luc reporter gene, pcDNA-HA or pcDNA3-HA-4CRU of Ahnak (amino-acid residues 4105–4633). SBE indicates Smad-binding element. Cells were incubated in the absence or presence of 10 ng/ml TGFβ for 20 h (**d**) or 6 h (**e**) in serum-free media. Then, cells were harvested for measurement of luciferase and β-galactosidase activities according to the manufacturer's protocol (Promega, Madison, WI, USA). Transfection efficiencies were normalized by measuring β-galactosidase activities. Data are means±s.e. of values from three independent experiments.

To validate the effect of Ahnak on TGFβ-mediated cell signaling, we examined Smad3 phosphorylation in MEFs from wild-type and Ahnak^−/−^ mice. Stimulation of Ahnak^−/−^ MEFs with TGFβ resulted in significantly decreased Smad3 phosphorylation compared to that with wild type (Figure 2a). We explored whether Ahnak protein is translocated to nucleus with R-Smad. NIH3T3 cells expressing green fluorescent protein (GFP)-4CRU of Ahnak were stimulated with TGFβ and then analyzed for nuclear localization of Ahnak and Smad2/3. The result indicated that GFP-4CRU of Ahnak is localized in cytoplasm in resting cells but moves to the nucleus with Smad2/3 after TGFβ stimulation (Supplementary Figure 2A). To verify the nuclear localization of the endogenous Ahnak protein on TGFβ stimulation, we used mouse primary smooth muscle cells, which contains a high level of Ahnak protein.^10^ Treatment of smooth muscle cells with TGFβ resulted in increase in the levels of nuclear Ahnak protein and Smad2/3 (Supplementary Figures 2B and C). We next questioned whether Ahnak protein is required for translocation of Smad3 in MEF cells. Wild-type MEFs showed a nuclear translocation of Smad3 in response to TGFβ (Figure 2b left), whereas Ahnak^−/−^ MEFs failed to stimulate Smad3 nuclear translocation (Figure 2b right) suggesting that Ahnak protein has a key role in Smad3 nuclear translocation.

It has been well established that TGFβ modulates cell cycle arrest through downregulation of c-Myc.^11,12^ We examined whether Ahnak regulates the initiation of c-Myc transcription. To evaluate the transcriptional effect of Ahnak, we used pBV-Luc-c-Myc promoter, a reporter construct containing c-Myc promoter region (-1073 to -423).^14^ NIH3T3 were co-transfected for 48 h with the c-Myc promoter–reporter plasmid with expression plasmids for 4CRU, NH2-terminal region or COOH-terminal region of Ahnak and then assayed for luciferase activity (Figure 2c). The expression of NH2- (NT) and COOH-terminal (CT) regions of Ahnak in NIH3T3 cells had no inhibitory effect on the activation of c-Myc promoter reporter, whereas the expression of 4CRU of Ahnak markedly inhibited c-Myc promoter–reporter activity (Figure 2c). The result suggested that Ahnak is involved in the regulation of c-Myc expression.

To investigate the functional effect of 4CRU of Ahnak in the TGFβ signaling, we next tested whether the overexpression of 4CRU of Ahnak has a positive effect in TGFβ-induced transcription. To avoid any interference of other DNA-binding cofactors, we used SBE-luc plasmid with synthetic SBEs to measure TGFβ-mediated stimulation. As shown in Figure 2d, the SBE-luc activity is increased 8- to 10-folds in the presence of 4CRU of Ahnak alone, and increased 75- to 90-folds in the presence of both 4CRU of Ahnak and TGFβ stimulation. To further verify the effect of Ahnak–Smad3 complex on the activation of SBE-luc reporter, we analyzed the activity in MEFs isolated from wild-type and Ahnak^−/−^ mice. As shown in Figure 2e, the SBE-luc activity in Ahnak^−/−^ MEF cells is significantly lower than that seen in wild-type MEFs. For functional verification of Ahnak in Smad-dependent transcriptional expression, we carried out chromatin immunoprecipitation experiments. TGFβ-induced nuclear localization of Ahnak resulted in increased binding of phosphoSmad3 to promoter loci of c-Myc and Smad7, suggesting that Ahnak regulates the expression of c-Myc and Smad7 as Smad3 target genes (Supplementary Figure 3A). These results suggest that Ahnak interacts with R-Smad and activates SBE, leading to potentiation of TGFβ signaling.

### Ahnak protein interacts with Smad7 inhibiting the interaction of Smad7 with TGFβRI

**Figure 3:**
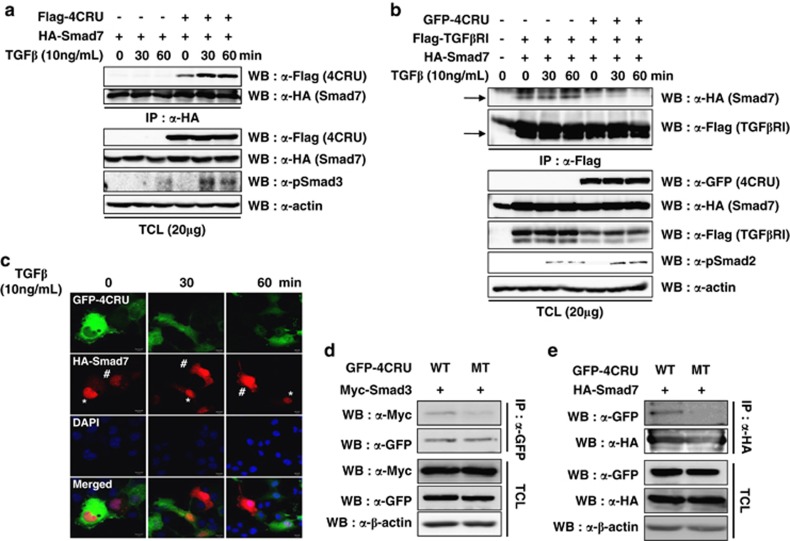
Interaction of Ahank with Smad7. (**a**) Interaction of Ahnak and Smad7. COS7 cells were transfected with Flag-4CRU of Ahnak and HA-Smad7. After serum starvation for 16 h, cells were stimulated without or with 10 ng/ml of TGFβ for indicated time. Cell lysates were immunoprecipitated (IP) with antibody to HA for Smad7 and then the immune complex was subjected to western blot (WB) analysis with antibodies to Flag and HA. (**b**) Ahnak disrupts the interaction of TGFβ receptor I (TGFβRI) with Smad7. COS7 cells were transfected with GFP-4CRU of Ahnak, Flag-TGFβRI and HA-Smad7. After serum starvation for 16 h, cells were stimulated without or with 10 ng/ml of TGFβ for indicated time. Cell lysates were IP with antibody to Flag for TGFβRI and then the immune complex was subjected to WB analysis with antibodies to HA and Flag. (**c**) Ahnak blocks the TGFβ-induced translocation of Smad7 from the nucleus to the cytoplasm. COS7 cells expressing GFP-4CRU of Ahnak (marked with asterisk, green) and HA-Smad7 (red) were stimulated with 10 ng/ml of TGFβ for indicated time and stained with antibody to HA and 4′-6-diamidino-2-phenylindole (DAPI; blue). TRITC-conjugated goat anti-mouse IgG was used as the secondary antibody for staining HA and analyzed by confocal microscopy. Representative images were shown. Scale bar: 10 μm. (**d**) COS7 cells were co-transfected with GFP-tagged wild-type (WT) or mutant (MT) 4CRU and Myc-tagged Smad3. Cell lysate were subjected to IP with antibody against GFP, and then performed WB analysis with indicated antibodies. (**e**) COS7 cells were co-transfected with GFP-tagged WT or MT 4CRU and HA-Smad7. Cell lysate were subjected to IP with antibody against HA, and then performed WB analysis with indicated antibodies. TCL, total cell lysate.

MH2 domain is conserved among R-Smads as well as in co-Smad and I-Smads. Therefore, we examined whether Ahnak protein interacts with I-Smad directly through MH2 domain. COS7 cells were transfected with HA-Smad7 and 4CRU of Ahnak. Stimulation of COS7 cells with TGFβ resulted in increased binding of Smad7 with CRUs of Ahnak protein (Figure 3a). To further dissect the nature of protein–protein interaction, Flag-TGFβRI and HA-Smad7 were co-transfected into COS7 cells with or without 4CRU of Ahnak and subjected to co-immunoprecipitation with antibody to Flag. Overexpression of Ahnak resulted in significantly diminished interaction of Smad7 with TGFβRI (Figure 3b). These results indicate that the presence of Ahnak protein leads to disruption of Smad7–TGFβ receptor complex, interfering with their activity. It is well known that Smad7 is localized in the nucleus in the absence of TGFβ stimulation.^15^ The Smad7 protein is exported to cytoplasm with Smurf, an E3 ligase after TGFβ stimulation, and it subsequently binds to TGFβRI on plasma membrane leading to degradation of the receptor by Smurf.^16,17^ We investigated whether the nuclear export of Smad7 is regulated by expression of Ahnak protein. GFP-4CRU of Ahnak and HA-Smad7 were co-tranfected into COS7 cells and then stimulated with TGFβ. Nuclear exporting of Smad7 were observed in COS7 cells expressing only HA-Smad7 (indicated by # in Figure 3c), whereas the exporting was significantly inhibited in COS7 cells expressing both HA-Smad7 and GFP-4CRU of Ahnak protein (indicated by * in Figure 3c). These results indicate that Ahnak protein potentiates TGFβ-mediated cell signaling through the concerted effects of activation of R-Smad and interference of I-Smad activities.

We found a mutant form of Ahnak from a cervical cancer tissue: valine^4607^ of wild type is converted into methionine^4607^ (Supplementary Figure 4A). To analyze the interaction of the mutant form with Smad3 or Smad7, we transfected wild-type or mutant 4CRU into COS7 cells in the presence of Smad3 or Smad7 and then performed co-immunoprecipitation experiment with antibodies against GFP for 4CRU or HA for Smad7. Interaction of mutant 4CRU with Smad3 or Smad7 was weaker than that of wild type (Figures 3d and e). We next validated the function of mutant 4CRU of Ahnak in expression of CDK inhibitor, p21^waf^. Expression of mutant form into COS7 cells failed to regulate p21^waf^ expression compared with that of wild type (Supplementary Figure 4B). These results with mutant 4CRU of Ahnak indicate the specificity of interaction between 4CRU and R-Smad or I-Smad. However, function of mutant 4CRU of Ahnak in cervical cancer needs to be determined.

### Ahnak protein inhibits cell proliferation through controlling cell cycle arrest

**Figure 4:**
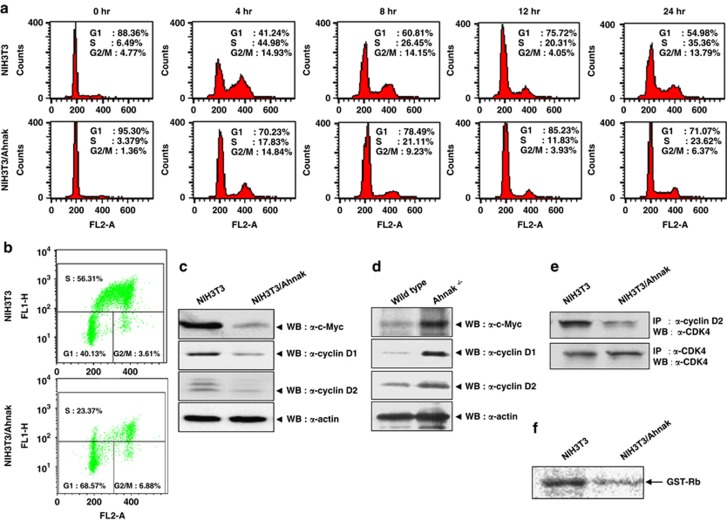
Expression of c-Myc, cyclins and CDK inhibitors (p21^Waf/Cip^ and p27^Kip1^) in NIH3T3/Ahnak. (**a**) Cell cycle analysis was performed using synchronized NIH3T3 and NIH3T3/Ahnak cells. The cells were harvested, stained with propidium iodide (PI) and applied to a Becton-Dickinson FACScalibur equipped with a single 488 nm argon laser. Fractions of G0/G1, S and G2/M were quantified using CELLQuest (Becton-Dickinson). (**b**) The cells were pulsed with bromodeoxyuridine (BrdU) for 3 h and then stained with anti-BrdU antibody and PI followed by fluorescence-activated cell sorting (FACS) analysis. (**c**) Cell lysates of NIH3T3 and NIH3T3/Ahnak were subjected to SDS–polyacrylamide gel electrophoresis (PAGE) and immunoblot analysis with antibodies to c-Myc, cyclin D1/D2 or actin. (**d**) Lysates of MEF cells from wild-type and Ahnak^-/-^ mice were subjected to SDS–PAGE and immunoblot analysis with antibodies to c-Myc, cyclin D1, cyclin D2 or actin. (**e**) The cell lysates were immunoprecipitated (IP) with antibodies to cyclin D2 and then analyzed by immunoblotting with antibody to CDK4. (**f**) Immune complex with antibody against cyclin D2 was subjected into *in vitro* kinase assay with GST-Rb as a kinase substrate. Immune complex kinase assay is described in Materials and methods. WB, western blot.

It has been well established that TGFβ has anti-proliferation and cytostatic effects on epithelial cells.^11,12^ To evaluate the function of Ahnak in cell growth, we established a line of NIH3T3 cells stably expressing 4CRU of Ahnak (NIH3T3/Ahnak). The growth rate of the NIH3T3/Ahnak cells was significantly lower than that of parental cells during the exponential growth phase (Supplementary Figure 5). To evaluate the effect of Ahnak on cell cycle, we tested the status of cell cycle in synchronized NIH3T3 and NIH3T3/Ahnak cells by fluorescence-activated cell sorting analysis with propidium iodide or bromodeoxyuridine staining and immunoblot analysis for cell cycle regulators. Both cell lines were synchronized with the double thymidine block and then released into S phase with serum. The result clearly showed that the overexpression of 4CRU of Ahnak in NIH3T3 cells led to accumulation of cells in G0/G1 phase (71%) compared with parental cells (55%) when examined 24 h after the release from the double thymidine block (Figure 4a). To clarify the ambiguous distribution of cells in S phase in fluorescence-activated cell sorting analysis, the cells were subjected to bromodeoxyuridine staining. The expression of 4CRU of Ahnak resulted in clear decrease in S phase entry (Figure 4b).

We next analyzed the expression levels of various effectors involved in cell cycle progression in both groups of cells. The expression of c-Myc and cyclin D1/D2 expression levels were significantly reduced in NIH3T3/Ahnak cells (Figure 4c). We also attempted to evaluate the effect of loss-of-function of Ahnak protein with Ahnak^−/−^ MEF cells. As expected, Ahnak^−/−^ MEF cells showed upregulated c-Myc and cyclin D expression (Figure 4d). These results indicated that Ahnak protein has an important role in cell cycle progression and proliferation through the regulation of c-Myc and cyclin D expression in response to TGFβ. Moreover, we next investigated the expression of the CDK inhibitors p21^Waf/Cip^ and p27^Kip1^, which are thought to specifically bind to cyclin–CDK complexes and proliferating cell nuclear antigen (PCNA), thereby serving as potent growth inhibitors of cell cycle progression.^11,12,18,19^ The expression levels of p21^Waf/Cip^ and p27^Kip1^ were increased in NIH3T3/Ahnak cells (Supplementary Figure 6A), indicating that G0/G1 arrest by Ahnak may be caused by not only the reduction of c-Myc and D-type cyclins but also the induction of p21^Waf/Cip^ and p27^Kip1^ expression.

To verify our conclusion, we examined the interaction of cyclin D with CDK4 in NIH3T3 and NIH3T3/Ahnak cells. Lysates were subjected to immunoprecipitation with antibodies against cyclin D2 or CDK4. Although total cell lysates contained similar amounts of CDK4, the amount of CDK4 in co-immunoprecipitated complex with cyclin D2 was markedly decreased in NIH3T3/Ahnak cells compared with parental cells (Figure 4e). Next, we investigated the activity of CDK4 in the immunoprecipitated complex with cyclin D2 in terms of *in vitro* GST-Rb phosphorylation. The immunoprecipitated CDK4 from NIH3T3/Ahnak cells showed a lower activity for GST-Rb protein phosphorylation compared with the immune complex from parental cells (Figure 4f). In addition, more CDK4 was in complex with CDK inhibitors, p21^Waf/Cip^ and p27^Kip1^ in NIH3T3/Ahnak cells compared with parental cells (Supplementary Figure 6B). Taken together, these results suggest that the mechanism of G0/G1-arrest induced by Ahnak may occur through the inhibition of CDK4 activity and dephosphorylation of Rb protein.

**Figure 5:**
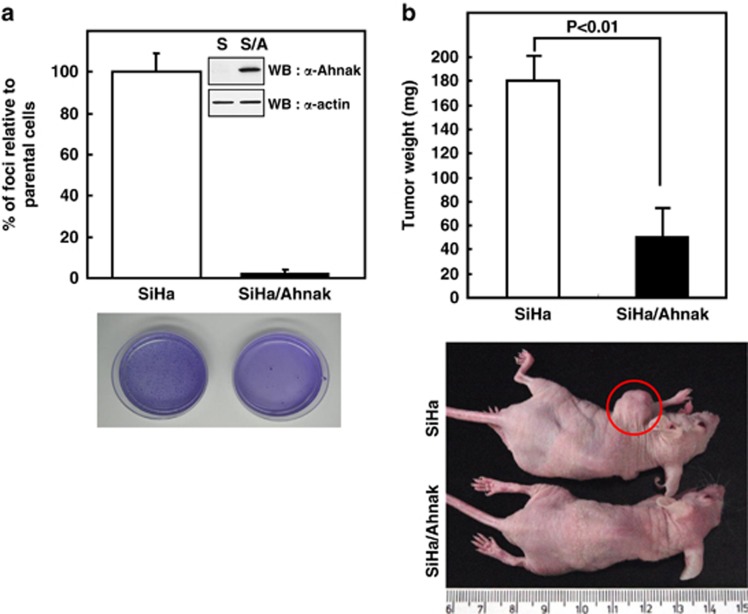
Retarded cellular proliferation of Ahnak cells. (**a**) SiHa and SiHa/Ahnak cells (5 × 10^3^) were suspended in 0.335% (w/v) agar/enriched medium solution and the mixture was overlaid onto a 0.5% (w/v) agar/enriched McCoy's medium. Cultures were scored for growth in the 0.335% agar layer after 20–25 days of incubation. The foci were visualized by staining with crystal violet. Data are means±s.e. of values from three independent experiments. (**b**) Parental SiHa and SiHa/Ahnak (1 × 10^7^) cells were subcutaneously injected into athymic nude mice (BALB/c-nu/nu, Charles River Co., Yokohama, Japan; *n*=8). Tumor mass was measured as described in Materials and methods. Data are means±s.e. of values from eight athymic nude mice. WB, western blot; S, SiHa cells; S/A, SiHa cells expressing 4CRU of Ahnak.

It has been established that a subset of tumor-suppressor proteins interacts with or negatively regulates the cell cycle machinery.^20^ We thus investigated whether Ahnak protein acts as a tumor suppressor. To test the gain-of-function of Ahnak in the inhibition of tumor growth, we established SiHa cells expressing 4CRU of Ahnak (SiHa/Ahnak).^21,22^ We measured oncogenic properties of SiHa and SiHa/Ahnak cells using colony-forming assay and tumor mass formation in nude mice. As shown in Figure 5a, control SiHa cells showed a much higher colony-forming activity on a soft agar than SiHa/Ahnak cells. Moreover, upon injection into athymic nude mice, the tumorigenic activity of SiHa/Ahnak cells was shown to be much lower than that of parental SiHa cells (Figure 5b). Our results are consistent with that Ahnak has a tumor-suppressive activity.

### Analysis of Ahnak knockout mice and tumor formation

**Figure 6:**
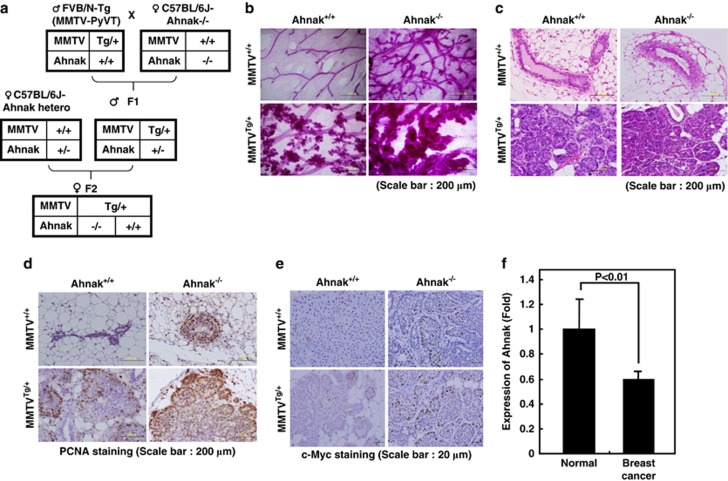
Tumor-suppressive effect of Ahnak. (**a**) Mating strategy for making mammary gland-specific expression of middle T antigen in Ahnak^-/-^ mice. (**b**) Whole mounted mammary glands from 6-week-old female virgin mice showed ductal morphology. (**c**) Benign mammary epithelium (upper panel) and malignant mammary tumor (lower panel) in 6-week-old female virgin mice were subjected to hematoxylin and eosin staining. (**d** and **e**) Mammary gland tissues from 6-week-old female virgin mice were stained with antibodies to PCNA (**d**) and c-Myc (**e**). (**f**) Ahnak expression level of human normal and breast cancer tissues.

To verify the function of Ahnak in tumor-suppressive activity, we crossed mice carrying the mammary gland specifically expressed polyomavirus middle T antigen (MMTV-PyVT) with Ahnak^−/−^ mice,^23^ and MMTV^Tg/+^Ahnak^−/−^ and MMTV^Tg/+^Ahnak^+/+^ mice were generated from mating of female MMTV^Tg/+^Ahnak^+/−^ mice with male MMTV^+/+^Ahnak^+/−^ mice (Figure 6a). Whole-mount preparation of the mammary gland showed significantly accelerated hyperplasia in MMTV^Tg/+^Ahnak^−/−^ mice compared with that in MMTV^Tg/+^Ahnak^+/+^ mice at 6 weeks of age (Figure 6b). Moreover, the epithelial layer of MMTV^+/+^Ahnak^−/−^ in the ducts of mammary gland was aberrantly thickened compared with that of MMTV^+/+^Ahnak^+/+^, although carcinogenesis was not as yet initiated (Figure 6c, upper panel). More malignant mammary tumor was seen in MMTV^Tg/+^Ahnak^−/−^ compared with MMTV^Tg/+^Ahnak^+/+^ (Figure 6c, lower panel). The expression of PCNA, a cell proliferation marker, was significantly increased in MMTV^Tg/+^Ahnak^−/−^ compared with the levels in MMTV^Tg/+^Ahnak^+/+^ (Figure 6d). The result could be confirmed by counting the number of PCNA-positive cells from histology slides in each group (Supplementary Figure 7). Furthermore, in wild-type mammary glands, the majority of epithelia and fibroblasts in ductal region were positive for pSmad2/3 (Supplementary Figure 8). In Ahnak^−/−^ mice, most of the epithelia in all ductal regions contained low levels of pSmad2/3 (Supplementary Figure 8). The result indicates that the R-Smad signaling pathway was less active in Ahnak^−/−^ mice. Next, we validated the concept on Ahnak-dependent c-Myc regulation in this tumor model. Expression of c-Myc in mammary gland tissues of Ahnak^−/−^ was significantly increased compared with that of Ahnak^+/+^ (Figure 6e). The result strongly indicates that Ahnak regulates c-Myc protein leading to controlling tumor growth.

To analyze loss of function of Ahnak in tumor, we also analyzed Ahnak expression level in tissues from breast cancer patients. Expression of Ahnak protein was decreased in breast cancer cells (Figure 6f). Moreover, we analyzed microarray data from Oncomine data base and found that Ahnak expression was significantly inhibited in malignant breast neoplasms compared with epithelial and common neoplasms (Supplementary Figures 9–11). These results suggest that Ahnak may be a tumor suppressor and that its deficiency increases breast cell proliferation and tumor development in the mice.

## Discussion

TGFβ signaling is mediated by modification of their receptor and interaction of Smad with regulatory proteins.^12,13^ Phosphorylation of TGFβ type I receptor is mediated by TGFβ type II receptor, and the phosphorylated receptor serves as the binding site for adaptor proteins as well as the inhibitory Smad7 and Smurf, which facilitate ubiquitination and degradation of TGFβ receptor.^24^ The other adaptor protein is FYVE domain containing SARA protein, which stabilizes phosphorylation of R-Smad interacting with TGFβ receptor.^25^ Indeed, proteins interacting with TGFβ receptor including Smad proteins have critical roles in TGFβ signaling for tissue development, homeostasis and metastasis.^26^ We showed that Ahnak protein interacts with MH2 domain of R-Smad and I-Smad proteins leading to regulation of nuclear localization of both proteins (Figures 1 and 3). Rather, Ahnak protein appears to have dual functions for regulation of TGFβ signaling. One is interaction with R-Smad protein (Figure 1) and the other is inhibition of I-Smad, Smad7 activity (Figure 3). We thus propose that Ahnak protein-mediated concerted controls for the activity of TGFβ receptor and of Smad allow the regulation in TGFβ signaling and cytostatic effect on cell growth. Cellular function of TGFβ signaling is regulation of epithelial-to-mesenchymal transition (EMT) resulting in cancer metastasis.^13^ We asked whether Ahnak protein can regulate TGFβ-induced EMT process. We found that EMT phenotype and marker genes were remarkably reduced in HaCaT human keratinocyte cells depleting Ahnak protein in response to TGFβ (data not shown). However, detailed function of Ahnak-dependent TGFβ activation in EMT needs for extensive studies.

A portion of Smad3 was phosphorylated and was detectable in the nucleus in Ahnak knockout MEF (Figures 2a and b) indicating that Ahnak-independent Smad3 phosphorylation and nuclear translocation of Smad3 are also possible to a certain extent. We found that Ahnak protein also binds to Smad4 (Figure 1h). To validate the function of interaction of Ahnak with Smad4, we used MDA-MG-468 cells deficient of Smad4. The cells were transfected with SBE-Luc, and pcDNA3-HA-4CRU of Ahnak (amino-acid residues 4105–4633) or Flag-Smad4. Stimulation of MDA-MG-468 cells with ectopically expressed 4CRU with TGFβ failed to activate SBE-Luc, whereas add-back expression of Smad4 showed an enhanced SBE-Luc activity (Supplementary Figure 12). These results indicate that 4CRU of Ahnak does not function as a co-Smad, although 4CRU directly interacts with Smad4. We also found that Ahnak protein failed to interact with TGFβ receptor or with PPM1A as a Smad phosphatase (data not shown).^27^ These results indicate that Ahnak protein neither acts as co-Smad function nor directly regulates R-Smad phosphorylation. The mechanisms and function of Ahnak-independent phosphorylation and nuclear translocation of Smad3 remain to be elucidated.

It has been well established that TGFβ signaling induces c-Myc downregulation in various cell types and the c-Myc has a critical role in the cell proliferation and development.^11, 12, 13,24^ Our results clearly demonstrated that Ahnak protein interacts with R-Smad and I-Smad through MH2 domain interaction, leading to the potentiation of TGFβ signaling cascades including c-Myc downregulation (Figures 1 and 2). Ahnak^−/−^ MEFs showed a higher growth rate than wild-type MEF cells through c-Myc upregulation (Figures 1a and 4d). Conversely, NIH3T3 cell lines stably overexpressing CRUs of Ahnak showed growth retardation with cell cycle arrest at G0/G1 stage and c-Myc downregulation (Figure 4 and Supplementary Figure 5). However, spontaneous carcinoma was not seen in Ahnak^−/−^ mice. Because the role of TGFβ in tumorigenesis is apparent under condition of oncogenic stress, we introduced MMTV-PyVT into Ahnak^−/−^ mice. Importantly, MMTV^Tg/+^Ahnak^−/−^ showed significant hyperplasia in mammary gland compared with that in MMTV^Tg/+^Ahnak^+/+^ (Figures 6b and c). These observations strongly suggest that Ahnak protein acts as negative regulator of c-Myc expression and thus as a tumor suppressor.

Although c-Myc protein is a potent oncogenic protein by itself, its transforming activity can be potentiated by collaboration with other oncogenic proteins. For example, collaboration of Ras with c-Myc is known to be critical for cellular transformation.^28^ It has been reported that Ras-mediated Myc stabilization results from activation of the sequential cascade of Ras-PI3-kinase-PKB.^28^ Thus, c-Myc and Ras network stimulates cyclin D-CDK4 pathway leading to cellular transformation. A recent report showed that overexpression of Ras resulted in downregulation of Ahnak protein expression.^29^ Our results in this report shed a light to our conclusion on Ahnak-dependent c-Myc downregulation and cell cycle arrest. In normal growth, Ahnak controls c-Myc expression, whereas transformed cells undergone Ras activation may downregulate expression of Ahnak protein, which in turn induces increased c-Myc expression resulting in cellular transformation.

It has been reported that TGFβ-mediated cell signaling has important roles in development of breast cancer.^30, 31, 32, 33, 34^ Expression of Ahnak protein was decreased in breast cancer tissues. In analysis of Oncomine database, Ahnak protein expression was shown to be decreased in malignant breast neoplasms consistent with our results (Figure 6f and Supplementary Figure 9). Analysis of human tumor specimens revealed that loss of Ahnak was strongly associated with hormone receptor-negative breast cancer, suggesting that this type of tumors heavily relies on Ahnak regulation of signaling pathways (Supplementary Figure 10). Significantly, many of the analyzed tumors were the ‘triple-negative (basal-like) breast cancers' (estrogen receptor (ER), progesterone receptor (PR) and erbB2 negative) that represent basal and mesenchymal mammary malignancies (Supplementary Figure 11).^35^ These tumors tend to be the most aggressive and represent a challenge to treat, and cytotoxic chemotherapy is currently the only systemic treatment option. Loss of Ahnak may permit uncontrolled tumor growth and cell cycle regulators. Thus, loss of Ahnak in hormone receptor-negative breast cancer may be considered as a marker and a target for designing future therapeutic strategies that will take into account the particular biology of this aggressive tumor subtype. These results indicate that Ahnak protein acts as novel tumor suppressor regulating cell proliferation. Therefore, controlling Ahnak activity or expression will be a viable potential tumor therapy.

**Figure 7:**
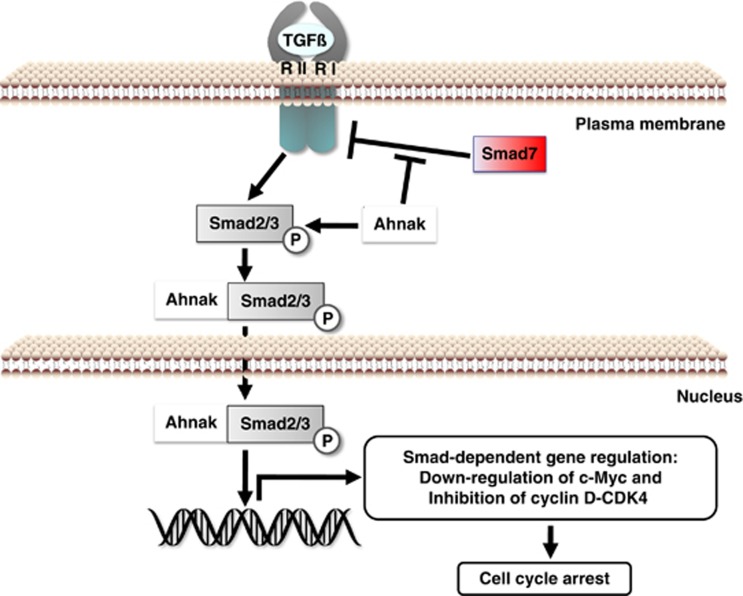
Putative model of Ahnak-dependent activation of TGFβ signaling. Ahnak protein interacts with R-Smad proteins for activation of R-Smad-dependent transcriptional regulation and with I-Smad for inhibiting I-Smad-mediated pathway, resulting in the downregulation of c-Myc and cyclin D protein. Downregulation of cyclin D initiates a series of events leading to the inactivation of CDK4 and dephosphorylation of Rb protein, resulting in cell cycle arrest at the G0/G1 stage. Thus, Ahnak serves as novel tumor suppressor through potentiation of TGFβ signaling.

In conclusion, we found that Ahnak protein interacts with R-Smad proteins through MH2 domain and Ahnak protein colocalizes with Smad3 in the nucleus of NIH3T3 cells in response to TGFβ. Moreover, Ahnak protein attenuates inhibitory Smad7 activity on TGFβ signaling. These sequential events in TGFβ signaling showed that Ahnak potentiates the transcriptional activity of R-Smad, leading to cell cycle arrest (Figure 7). Taken together, we conclude that Ahnak is involved in the regulation of cell growth and acts as novel tumor suppressor through potentiation of TGFβ signaling.

## Materials and methods

### Isolation of MEFs

MEFs were isolated from embryos in E 13.5. The uterus was dissected out from mouse and rinsed with phosphate-buffered saline. After isolation of each fetus in phosphate-buffered saline, all of the organs and head were removed to isolate only trunk. Each trunk was finely minced by ejecting with 10 ml syringe. The mixture of cells and small tissue mass were incubated with 5 ml trypsin-EDTA at 37 °C in shaking incubator for 30 min. Fetal bovine serum was added and then followed by addition of 5 ml of fresh medium (Dulbecco's modified Eagle's medium, 10% fetal bovine serum). The cells were resuspended and transferred to 150 mm culture dish. The MEFs were grown until 70–80% confluence. Second- or third-passage MEFs were used in all experiments.

### Yeast two hybrid screening

Two-hybrid screening in yeast was performed by the interaction trap cloning method, which is often referred to as the LexA two-hybrid system. One repeat unit (amino acids 3988–4129) of human Ahnak was cloned in-frame with the LexA DNA-binding domain. Screening was done using a HeLa cell cDNA library in pB42AD. To identify the Ahnak-binding domain of human Smad2, the cDNA of encoding full length, MH1, Linker, MH2 domain of hSmad2 were subcloned into the LexA-activation domain fusion vector pB42AD. EGY48 yeast cells were transformed with the prey plasmid, the bait plasmid and the p80p-lacZ by the lithium acetate transformation method. Yeast cells containing bait plasmid and lacZ reporter plasmid were transformed with 100 μg of library plasmid and plated on glucose medium lacing tryptophan, histidine and uracil, to select for the presence of all three plasmids. 2 × 10^6^ colonies were obtained. Yeast transformants were selected for the reporter gene LEU2 and lacZ gene transcription under the control of multiple LexA operators. The interacting cDNA clones were rescued from the selected yeast transformants.

### Measurement of cellular growth rate

To measure the growth rate of the NIH3T3 cells, which constitutively express the 4CRU of Ahnak, 3 × 10^5^ cells were seeded into 100 mm dishes and cultured for 5 days. To measure the MEFs growth rate, wild-type and Ahnak knockout MEF cells (2.5 × 10^3^) were seeded into wells of 96-well plates and cultured for 7 days. The cultures were replenished with fresh media every other day. Cells from duplicate wells were harvested with trypsin-EDTA daily and the total number of cells was determined by counting with a hemocytometer. The data are mean±s.e.m. of three independent experiments.

### Generation and identification of MMTV^Tg/+^Ahnak^−/−^ mice

Generation and characterization of Ahnak^−/−^ mice were described in previous report.^9,10^ FVB/N-Tg (MMTV-PyVT) 634Mul/J mice were purchased from the Jackson Laboratory (Bar Harbor, ME, USA).^14^ To obtain MMTV^Tg/+^Ahnak^−/−^ mice (mixed genetic background) at F2 generation, male MMTV^Tg/+^ mouse (FVB genetic background) was crossed with female Ahnak^−/−^ mouse (C57BL/6 genetic background). Male F1 MMTV^Tg/+^Ahnak^+/−^ mouse was crossed with female Ahnak^−/−^ mouse (C57BL/6 genetic background) again. All experiments on mammary cancer were performed using 6-week-old female F2 MMTV^Tg/+^Ahnak^+/+^ or MMTV^Tg/+^Ahnak^−/−^.

### Mammary gland whole mounts

Mammary glands were dissected, spread on glass slides, fixed overnight in Carnoy's fixative (six parts 100% EtOH, three parts CHCl_3_, one part glacial acetic acid) and stained with Carmine Alum (1 g carmine (Sigma, St Louis, MO, USA) and 2.5 g aluminum potassium sulfate (Sigma) in 500 ml distilled water). Photos were taken using BX51 light microscope (Olympus, Tokyo, Japan) equipped with a digital camera (DP71, Olympus).

### Histological analysis

Mammary gland tissue was fixed in 4% neutral buffered formalin and embedded in paraffin. Sections (5 μm) were routinely stained with hematoxylin (Sigma) and eosin Y (Sigma). For immunohistochemistry against PCNA, sections were hydrated, treated with 0.3% hydrogen peroxide in methanol for 15 min and proceeded mediated antigen retrieval with Tris-EDTA. Slides were blocked with 10% normal goat serum in 0.01 M phosphate-buffered saline for 30 min before anti-PCNA antibody binding for overnight at 4 °C. After HRP-conjugated secondary antibody binding, sections were subsequently exposed to Dako REAL EnVision Detection System, Peroxidase/DAB+, Rabbit/Mouse (Dako, Glostrup, Denmark). Images were taken through BX51 light microscope (Olympus) equipped with a digital camera (DP71, Olympus). The number of PCNA-positive cells was counted from the histology slides in mammary gland tissue of MMTV^+/+^Ahnak^+/+^, MMTV^+/+^Ahnak^−/−^, MMTV^Tg/+^Ahnak^−/−^ and MMTV^Tg/+^Ahnak^+/+^. In each experimental mice group, five slides of three animals were used.

### Statistics

Statistical analysis was performed with a two-tailed unpaired *t*-test. Data are expressed as means±standard deviations (s.d.) of values from three to five independent experiments. All western blot analysis in figures were representative of three independent experiments.
