# Supplementary material for: Ahnak functions as a tumor suppressor via modulation of TGFβ/Smad signaling pathway
Source: Oncogene. 2014 Mar 24;33(38):4675–84. doi: 10.1038/onc.2014.69 (PMC4180639; doi:10.1038/onc.2014.69)
Supplement: Supplementary Information [file onc201469x1.doc]

**Supplementary Figures and Legends**

**Supplementary Figure S1.** Body size of wild type and Ahnak^-/-^ mouse.

**Supplementary Figure S2.** Nuclear localization of Ahnak with Smad2/3. **A,** NIH3T3 cells were transfected with GFP-4CRU of Ahnak. Cells were stimulated with 10ng/mL of TGFβ for indicated time. The cells were fixed and stained with antibody to Smad2/3 (red) analyzed by confocal microscopy. DAPI (blue) represents nuclear staining. **B,** Smooth muscle cells (SMC) were stimulated with 10ng/mL of TGFβ for indicated time. The cells were stained with antibodies to Ahnak protein (red) and Smad2/3 antibody (green) and they were analyzed by confocal microscopy. **C,** Relative intensity of nuclear localization of Ahnak with Smad2/3 was quantitated from Figure S2B. Data are means ± standard deviation (SD) of nuclear localization of Ahnak with Smad2/3 and are representative of three independent experiments (P<0.0000001).


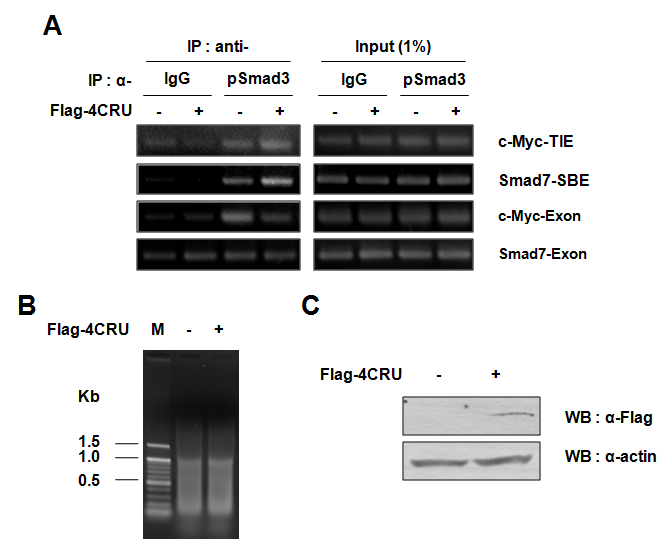


**Supplementary Figure S3.** **Chromatin immunoprecipitation (ChIP) with antibody to pSmad3 in the absence or presence of Ahnak protein. A,** Flag-4CRU-transfected HaCaT human keratinocyte cells were subjected to ChIP assay. Purified DNA was amplified by PCR. PCR primers were designed according to promoter sequences containing Smad3-binding sits of indicated target genes. SBE: Smad-binding element, TIE: TGF-β-inhibitory element, Exon: exon of indicated genes (negative control). **B,** DNA shearing (0.1-1Kb) in ChIP assay was presented via DNA electrophoresis. M indicates DNA size marker. **C,** Cell lysate was obtained after reverse cross-linking during ChIP assay and subjected to western blot analysis with indicated antibodies.


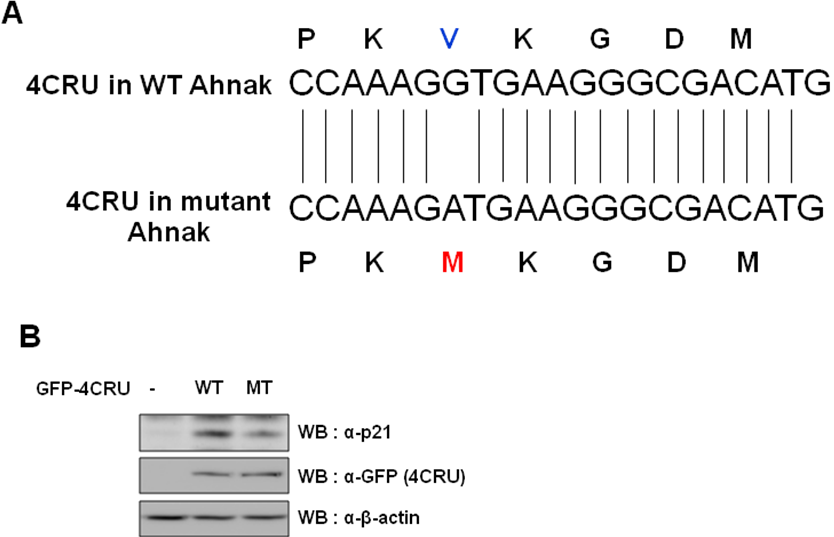


**Supplementary Figure S4. Effect of mutant 4CRU of Ahnak on** p21^waf^ expression. **A**, Single nucleotide mutation of Ahnak in cervical cancer. 4CRU of Ahnak means four central repeated units (AA 4105-4633) of Ahnak. **B**, Ahnak-dependent p21^waf^ expression. COS7 cells were transfected with HA-tagged 4CRU (WT) or mutant 4CRU (MT) of Ahnak. Cell lysates were analyzed with antibody to p21^waf^.


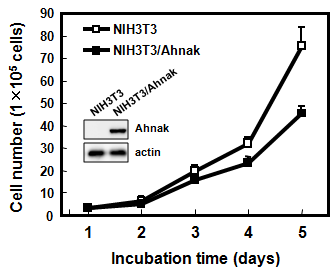


**Supplementary Figure S5.** Cell growth patterns of parental NIH3T3 and NIH3T3 expressing 4CRU of Ahnak (NIH3T3/Ahnak). Ectopic expression of 4CRU of Ahnak was confirmed by immunoblot analysis with antibody against Ahnak (inset).

**A**

**B**

**IP : α-CDK4**

**WB : α-CDK4**

**IP : α-CDK4**

**WB : α-p21**


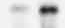

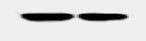

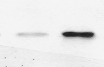


**IP : α-CDK4**

**WB : α-p27**

**WB : α-p21**


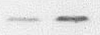

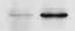


**WB : α-p27**

**WB : α-actin**


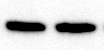


**WB : α-cyclin A**


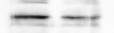

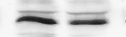


**WB : α-cyclin B**


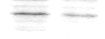


**WB : α-cyclin E**

**Supplementary Figure S6.** **A**, Expression of cell cycle-related proteins in NIH3T3 and NIH3T3 expressing 4CRU of Ahnak (NIH3T3/Ahnak). The cell lysates were separated with SDS-PAGE and analyzed by immunoblot with antibodies against cyclin A, cyclin B, cyclin E, p21^Waf/Cip^, p27^Kip1^ or actin. **B**, Interaction of CDK4 with p21^Waf/Cip^ or p27^Kip1^. The cell lysates were immunoprecipitated with antibodies to CDK4 and then analyzed by immunoblot with antibodies to CDK4, p21^Waf/Cip^, or p27^Kip1^.


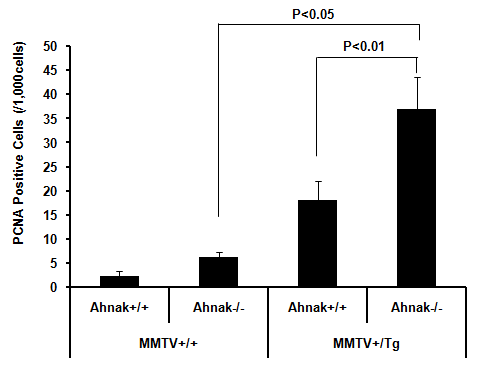


**Supplementary Figure S7.** The number of PCNA positive cells in mammary gland tissue of Ahnak^-/-^ mouse.

**
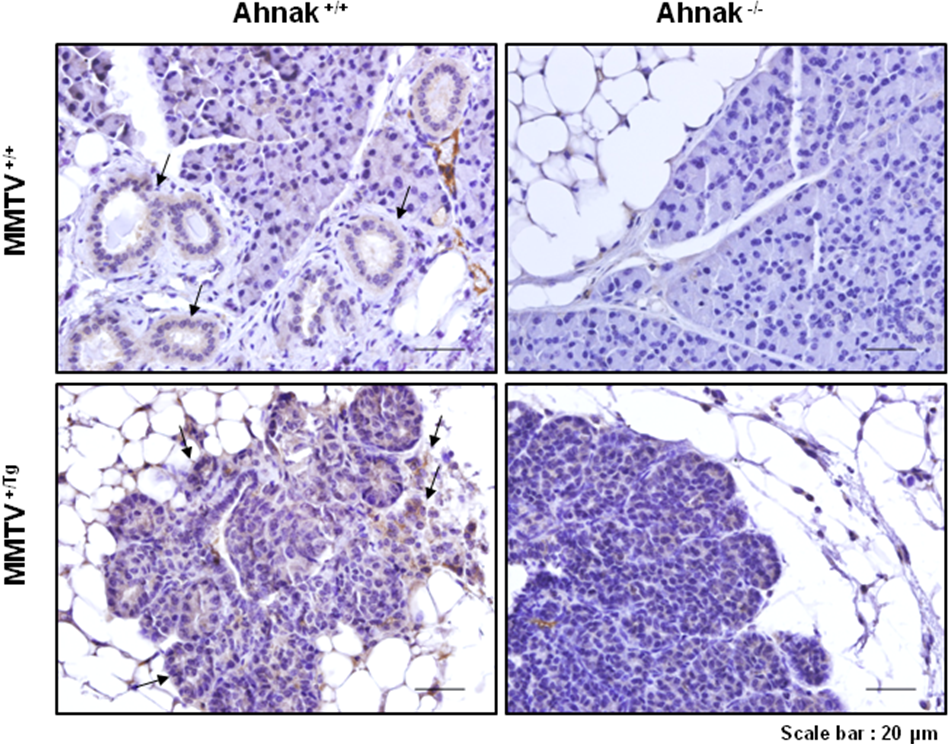
**

**Supplementary Figure S8.** Mammary gland tissues from 6-week-old female virgin mice were stained with antibody to pSmad2/3 (brown, arrow) and with hematoxylin (blue).

**Supplementary Figure S9.** Expression level of Ahnak in epithelial, common, and malignant breast neoplasm through Oncomine database.

**Supplementary Figure S10.** Estrogen receptor-dependent Ahnak expression in breast cancer tissues through Oncomine database.

**Supplementary Figure S11.** Expression level of Ahnak in various cancer tissues through Oncomine database.

**Supplementary Figure S12.** Function of Ahnak in MDA-MG-468 cells deficient Smad4. MDA-MG-468 cells deficient Smad4 were transfected with SBE-Luc, and pcDNA3-HA-4CRU of Ahnak (amino acid residues 4105-4633) or Flag-Smad4. Cells were incubated in the absence or presence of 10ng/mL TGFβ in serum-free media and then harvested for measurement of luciferase and β-galactosidase activities according to the manufacturer's protocol (Promega, Madison, WI). Transfection efficiencies were normalized by measuring β-galactosidase activities. Data are means ± S.E. of values from three independent experiments.

**Supplementary Table and Legend**

**Supplementary Table S1.** Summary of Ahnak-interacting proteins analyzed by yeast two hybrid.

**Supplementary Materials and Methods**

*Cell cultures*

Mouse embryonic fibroblasts (MEFs), COS7 cells and smooth muscle cells (SMC) were cultured at 37°C in an atmosphere of 5% CO_2_ in DMEM supplemented with 10% (v/v) FBS and 1% (v/v) antibiotic-antimycotic solution (GIBCO). NIH3T3 cells were cultured in DMEM supplemented with 10% calf serum.

*Antibodies*

Monoclonal antibodies against p27^Kip1^ and Rb were obtained from BD Transduction Laboratories (Palo Alto, CA). Monoclonal antibodies against cyclin D1, cyclin D2, cyclin A, cyclin B, cyclin D3, cyclin E, p21^Waf/Cip^, CDK4, Myc-tag (9E10), HA and GFP were purchased from Santa Cruz Biotechnologies (Santa Cruz, CA). Monoclonal antibody against Flag was purchased from Sigma. Polyclonal antibody against phospho-Smad2 (pSmad2) and monoclonal antibody against phospho-Smad3 (pSmad3) were obtained from Cell Signaling. Monoclonal antibody against proliferating cell nuclear antigen (PCNA) was purchased from Abcam. Monoclonal against BrdU antibody, Alexa Fluor® 488 conjugate was obtained from Molecular Probes.

*Establishment of NIH3T3 or SiHa cells stably expressing 4CRU of Ahnak*

NIH3T3 or SiHa cells (2×10^7^) in the log phase were harvested, washed with cold SMEB buffer, resuspended in 300μL of SMEB buffer (270mM sucrose, 7mM NaPO_4_, pH7.4 and 1mM MgCl_2_) containing pcDNA3.1 (Zeo)-4CRU (amino acid residues 4105-4633) and allowed to stand at room temperature for 15min. After electroporation, cells were then placed on ice for 10min, transferred to 150mm dishes containing culture media with 10% FBS and incubated in 5% CO_2_, at 37°C. The cells had stabilized, 200μg/mL of zeocin was added, and incubation continued for 20-25 days with medium changes every 3-4 days.

*Immunofluorescence staining*

COS7 cells were plated at a density of 3×10^4^ cells/well in 12-well plates on coverslips. The cells were transfected with 0.2μg of pEGFP-N1-4CRU of Ahnak, 0.2μg of pcDNA3-HA-Smad7. The cells were deprived of serum for 16hr, stimulated with or without 10ng/mL TGFβ (R&D Systems) for the indicated times, washed with cold PBS, fixed with 3.5% paraformaldehyde in PBS for 10min at room temperature, washed with PBS, and then permeabilized in 0.5% Triton X-100. Nonspecific sites were blocked by treating the cells with PBS containing 0.5% bovine serum albumin for 4hr at 4°C. The cells were incubated with primary antibody against HA in PBS overnight at 4°C, washed with PBS, incubated with the secondary antibody (TRITC-conjugated goat anti-mouse-IgG, KPL) for 40min at room temperature in the dark, and then mounted on glass slides using a drop of Aqua-Poly/mount. Images were recorded using a model LSM510 Meta confocal laser scanning microscope (Carl Zeiss).

*Cell cycle analysis*

1-2×10^6^cells were fixed in 70% ethanol for overnight at -20°C and collected by low speed centrifugation. After being washed twice with PBS, DNAs were stained with propidium iodide (PI, 50µg/ml) in the presence of 50µg/mL of RNase A (Sigma). Stained cells were then processed with a FACScalibur instrument and analyzed with Cell Quest software (Becton Dickinson).

*Colony formation assay in soft agar*

SiHa cells (5×10^3^) were suspended in 1.5mL of 0.335% (w/v) agar/α-MEM medium solution. Cells were overlaid onto a 0.5% (w/v) agar/enriched McCoy’s [McCoy’s 5A medium, 200μg/mL streptomycin, 200IU/mL penicillin, 0.44g/L sodium pyruvate, 80mg/L L-serine, 4mM L-glutamine, 25% FBS, 12.5% horse serum]. Cultures were scored for growth in the 0.335% agar layer after 20-25 days of incubation.

*In vivo tumor formation test*

To test the *in vivo* effect of Ahnak on the inhibition of tumor growth, we injected parental SiHa cells (1×10^7^) and SiHa cells expressing 4CRU of Ahnak (SiHa/Ahnak, 1×10^7^) subcutaneously into 8 week old athymic nude mice (male BALB/c-nu/nu, Charles River Co., Japan). Mice were maintained in air barrier units with constant temperature (25±1°C), humidity (60±5%) and a 12-hr light cycle. Mice were fed a normal diet (Lab Diet. Co. USA). Two weeks after inoculation, mice were sacrificed under methoxyfluorane anesthesia. Tumor tissues were collected from them and fixed in 10% neutralized buffered formalin. Tumor volumes were determined using caliper measurements of tumor length (L) and width (W) according to the formula (L x W x W)/2.

*Immuno-complex kinase assay*

For the immune complex kinase assay, cyclin D2-CDK complex on beads was incubated with 1μg of GST-Rb, 100μM ATP and 2μCi of [γ-^32^P] ATP in 30μl of kinase buffer. The kinase reaction was performed at 30°C for 30min and then terminated with 2x SDS-PAGE sample buffer. The reaction mixtures were resolved by SDS-PAGE analysis. Gels were stained with Coomassie Blue staining solution, dried under vacuum, and exposed to the BAS 2500 system for autoradiography.

*Luciferase Reporter Assays*

The division of Ahnak into N- (amino acid residues 1-500), 4CRU (amino acid residues 4105-4633), C1- (amino acid residues 4888-5634) and C2- (amino acid residues 5505-5890) domains, was based on a previously published report. The cDNA fragments were amplified by PCR and inserted into pcDNA3-HA vector. NIH3T3 cells were transfected with appropriate combination of the reporter (pBV-Luc 650bp c-Myc, c-Myc promoter region; -1073 to -423), various fragments of Ahnak (N-, C1- C2, and 4CRU), and control plasmids. The cells were cultured for 48hr after transfection, and luciferase and β-galactosidase activities were measured according to the manufacturer's protocol (Promega, Madison, WI). Transfection efficiencies were normalized against β-galactosidase activities. MEFs (4×10^4^/well) were seeded in culture dish and co-transfected with pSBE-Luc and β-galactosidase-expressing plasmid. The cells were stimulated with 10ng/mL of TGFβ for 6hr. Reporter assay was performed using Luciferase Assay System (Promega) according to manufacturer’s protocol.

*Chromatin immunoprecipitation (ChIP) assay*

10^6^ HaCaT human keratinocyte cells were seeded in 100mm culture dishes and transfected with plasmids expressing Flag or Flag-4CRU. 24hr after transfection, ChIP assay was performed using EZ ChIP™ Chromatin Immunoprecipitation Kit (Millipore) in accordance with manufacturer's protocol. Sonication was perfomed at 30% amplitude for 10s and the pulse interval was 1s. It was repeated 8 times on ice. The amount of genomic DNA was measured through optical density at 260nm and each 20μg of cell extract was reacted with 2μg of indicated antibodies for 12-16hr at 4°C. Precipitated DNA was purified using QIAquick PCR Purification Kit (Qiagen). DNA was eluted with 30μL of ddH_2_O and 2μL was used for PCR. FastStart Taq DNA Polymerase, dNTPack (Roche) was used for PCR with following primers :

c-Myc-TIE forward 5’– AGATTTCAGTAGCAGCCTTG – 3’

c-Myc-TIE reverse 5’– TTGCAGATCACTCTATTCAG – 3’

c-Myc-Exon forward 5’– TCCTGGCAAAAGGTCAGAGT – 3’

c-Myc-Exon reverse 5’– TGTCCAACTTGACCCTCTTG – 3’

Smad7-SBE forward 5’– ACAGACCCGGGAGGGGGTGG – 3’

Smad-7-SBE reverse 5’– ACCCCCCCTCCGGCCCTCTG – 3’

Smad7-Exon-forward 5’– TTTCTCCATCAAGGCTTTCG – 3’

Smad7-Exon-reverse 5’– CTGATGAACTGGCGGGTGTA – 3’

PCR product was analyzed with 1.5% agarose gel electrophoresis. DNA shearing was analyzed with 1% agarose gel electrophoresis after reverse-crosslinking in ChIP process. After sonication, 20~50μL of cell lysate was diluted equally by measuring OD_260_, reversely crosslinked, mixed with SDS-PAGE sample buffer, and subjected to western blot analysis with indicated antibodies for detection of ectopically expressed 4CRU.

*Western blot analysis*

To study on Smad3 phosphorylation, 105 WT or Ahnak KO MEFs were seeded in 6-well culture dishes and stimulated with 10ng/mL of TGF-β for 0, 5, 10, 20, 30, and 60m after 12-16h of serum starvation in DMEM serum-free medium. Cells were lysed with 150μL of 1X SDS-PAGE sample buffer for complete lysis including nucleus. After sonication (7% amplitude, 5s, 1s of pulse interval), lysates were boiled at 95°C for 5m. 50μL of each lysate was subjected to western blot analysis using indicated primary antibodies. Horseradish peroxidase (HRP)-conjugated goat anti-rabbit or mouse IgG (Santa Cruz, CA) was used as secondary antibodies for chemiluminescent imaging. Fold increase was calculated by assessing band intensity (pSmad3/Smad3) using Photoshop 7.0 software (Adobe).

NIH3T3 cells were lysed with lysis buffer containing 50mM Tris-HCl pH 7.4, 150mM NaCl, 10% Glycerol, 1% Triton X-100, 0.5% NP-40, 1mM EDTA, 1μg/mL leupeptin, 1μg/mL aprotinin, and 0.1μM AEBSF. Lysates were quantified with BCA assay kit (Pierce), mixed with SDS-PAGE sample buffer, and boiled at 95°C for 5m. Cell lysates were subjected western blot analysis using indicated antibodies.

*Co-Immunoprecipitation assay (Co-IP)*

Lysates (0.5-1.5mg) were mixed with indicated antibodies for overnight at 4°C and incubated with 30-40μL of protein G-Sepharose (Roche) for 2h at 4°C. Immune complex was washed with lysis buffer, boiled with 2X SDS-PAGE sample buffer at 4°C, and subjected to western blot analysis with indicated antibodies.
